# Thermo-Mechanical Optimization of Die Casting Molds Using Topology Optimization and Numerical Simulations

**DOI:** 10.3390/ma17092114

**Published:** 2024-04-30

**Authors:** Serouj Djabraian, Fabian Teichmann, Sebastian Müller

**Affiliations:** Institute of Casting Technology, Friedrich-Alexander-Universität Erlangen-Nürnberg, Dr.-Mack-Str. 81, 90762 Fürth, Germanyseb.mueller@fau.de (S.M.)

**Keywords:** topology optimization, thermal optimization, structural optimization, die casting, mold, cooling structures, heat sink, COMSOL multiphysics

## Abstract

Conventional cooling channels used in die casting molds exhibit significant drawbacks, resulting in extended cooling times for cast parts. Issues such as the formation of dirt, limescale, and corrosion substantially diminish the thermal efficiency of these channels, leading to challenges in achieving uniform cooling and potential quality issues. In response to these challenges, this study proposes Topology Optimization as a novel approach. It involves designing cooling structures through Topology Optimization to replace traditional cooling channels, incorporating both Discrete and Gaussian boundary conditions to optimize thermal efficiency. Additionally, Structural Topology Optimization is employed to ensure structural integrity, preventing deformation or yielding under high loads during the die casting process. Numerical analysis revealed superior thermal performance compared to conventional channels, particularly when subjected to Discrete and Gaussian boundary conditions. Furthermore, the application of the latter establishes conformal cooling and minimizes temperature gradients in the casting, reducing casting defects such as shrinkage porosity. These findings highlight the efficacy of Topology Optimization in addressing the challenges of traditional cooling methods, with wide-ranging implications for manufacturing processes utilizing permanent molds for shaping materials.

## 1. Introduction

Die casting is a well-established generative forming process, widely recognized in both academic research and industrial applications. This method facilitates the production of components characterized by complex geometries and uniform material properties. One pivotal aspect of die casting, which significantly impacts material properties, production speed, and overall efficiency, is the management of heat within the die casting molds [1,2]. The duration of cooling times is directly correlated with the solidification times, imposing an increased thermal load on the die. This, in turn, has a profound effect on the lifespan of the die [3]. Conventionally, heat is dissipated using cooling channels, with water typically serving as the coolant. However, in instances where cooling at or above water’s boiling point is necessary, oil may be employed as an alternative. The strategic placement of these cooling channels is crucial to the mold design, ensuring uniform and efficient cooling of the cast part. This is vital for maintaining both the economic viability and the quality of the final product [4].

Not only does the large cooling requirement of these channels contribute to increased operating costs, but it also leads to a gradual development of limescale, dirt, and corrosion over time. These can build up and block the cooling channels, reducing the overall heat transfer coefficient, thus hindering the total heat transfer and resulting in increased cooling times and energy use [5]. For reference, the thermal conductivity of limescale is in an order of magnitude lower than that of tool steel, which obstructs the heat flow from the working fluid to the mold. An examination of the impact of limescale accumulation on the heat transfer efficiency within tubular heat exchangers revealed that a limescale layer of 2 mm thickness results in an approximate 12.5% increase in energy consumption [6]. Furthermore, a separate investigation into the effects of limescale on heat transfer within injection molding processes, particularly those utilizing conformal cooling channels, determined that a 2 mm thickness of limescale significantly hampers heat dissipation. Consequently, this reduction in efficiency renders the advanced conformal cooling system only as effective as the less sophisticated conventional cooling system in terms of heat extraction capabilities [7].

In die casting, producing high-quality components during the casting process depends significantly on maintaining uniform cooling. Differences in temperature inside the casting can result in shrinkage porosity, a phenomenon that significantly compromises the structural integrity and the material properties of the casting. Shrinkage porosity develops when local variations in temperature lead to uneven cooling rates. Minimizing temperature variations throughout the casting is essential to enhance the overall mechanical characteristics, as well as for reducing the likelihood of porosity formation [8]. A recent investigation into the effect of conformal cooling inserts in the high-pressure die casting of AlSi9Cu3 demonstrated a 43% decrease in porosity when conformal cooling channels were designed in a die casting mold using the additive manufacturing technique known as Direct Metal Laser Sintering (DMLS) [9].

One promising method to address these challenges is the application of Topology Optimization. Topology Optimization is a computational design approach that aims to maximize the performance of structures within a defined design space, and it has many extensions and applications. Nowadays, Topology Optimization is used to enhance heat sinks design used in different applications with superior thermal efficiency [10,11,12,13,14,15,16,17,18]. However, with the significant degree of design freedom that Topology Optimization provides, complex structures can be explored and optimized to achieve particular technical goals. Most of the time, these complex structures cannot be manufactured using conventional manufacturing methods such as machining. In a recent experimental study, geometrically complex 3D heat sinks for natural convection were manufactured using Investment Casting (IC) via 3D Stereolithography (SLA) in Brittania metal, which concluded that IC with the help of SLA is a very promising, low-cost, highly accurate method for fabricating metal parts generated through Topology Optimization. Furthermore, the study’s findings demonstrated that, in comparison to pin-fin heat sinks, the evaluated Topology Optimization heat sinks could consistently achieve the best heat dissipation performance [19]. In another recent experimental validation, similar 3D geometrically complex heat sinks were realized in an aluminum alloy using an additive manufacturing process, such as Selective Laser Melting (SLM), where the results of the study showed that the evaluated Topology Optimization heat sinks achieved better heat dissipation performance when compared to parametrically optimized heat sinks [12,20].

In the context of die casting molds, Topology Optimization can be used to generate optimized cooling structures, replacing the conventional circular cooling channels, ensuring efficient and uniform cooling of the casting throughout the mold, while enabling regulation of the local temperatures and cooling rates at different parts of the casting to control, reduce, or eliminate shrinkage porosity. Thermal regulation of local temperatures in the casting is realized by the application of Discrete and Gaussian probability distribution functions as boundary conditions to the Topology Optimization design domain, which minimizes the temperature gradients in the casting through controlled cooling. Consequently, this approach opens up the possibility of employing air as an alternative to traditional cooling fluids, addressing the issue of limescale formation, and providing a more sustainable cooling solution. However, several issues may unfold when using these optimized cooling structures, such as the influence of thermal contact resistance with the die casting mold, which hampers the heat conduction. Furthermore, estimation of the local heat transfer coefficient (HTC) at the outer surfaces of these cooling structures might be difficult because of its complex and non-streamlined geometry, since regions with flow recirculation will diminish the local heat transfer rate associated with locally low surface Nusselt numbers [21].

In order to compare the optimization efficiency of these cooling structures to that of the cooling channels, different Topology Optimization boundary conditions as well as different solid fraction (SF) constraints are considered throughout the study. Furthermore, the study extends to include fins as an additional case study, given their widespread use in numerous cooling applications. This comparison aims to evaluate the efficiency of the proposed cooling structures relative to simpler yet effective solutions that are commonly employed in various components. Multiple numerical heat transfer simulations are performed with simplified convective boundary conditions on the surfaces of the cooling channels, fins, and cooling structures with a total cooling time of 30 s, and the average temperature of the casting $T_{avg}$ is analyzed to compare the effectiveness of different cases. To ensure compatibility between the different investigated setups, AlSi10Mg was used as an aluminum alloy for the fins and the cooling structures in the numerical simulations, which is one of the most commonly used and well-established alloys in additive manufacturing, selected because of its exceptional mechanical properties and lightweight characteristics [22].

## 2. Methods and Computational Models

### 2.1. Method

**Figure 1:**
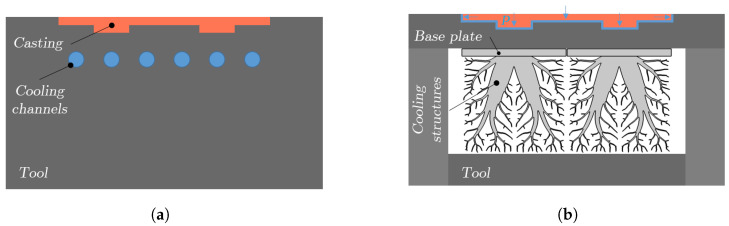
Two different types of die casting molds with internal cooling. (**a**) Conventional cooling channels. (**b**) Cooling structures.

Figure 1 provides a schematic comparison between traditional cooling channels and the novel cooling structures in a die casting mold. In Figure 1a, a series of cylindrical cooling channels are depicted, positioned at a specific distance from the casting to facilitate cooling. Conversely, Figure 1b illustrates cooling structures that are affixed to the mold through base plates. Given the varying cooling requirements of different parts of the casting, multiple cooling structures, each offering distinct cooling capabilities, can be arranged adjacent to one another or substituted with a single, larger cooling structure tailored to the casting’s geometry. Notably, the mold may experience bending due to the considerable pressure applied during the die casting process, particularly in regions where a significant cross-sectional area of the mold remains unsupported. In scenarios where bending is anticipated, supplementary support structures can be incorporated into the cooling structures’ design and secured to the mold with an additional base plate. This enhancement is vital for preventing displacement and deformation of the mold, ensuring its structural integrity and the dimensional accuracy of the castings. More about the incorporation of support structures into the cooling structures is discussed later.

**Figure 2:**
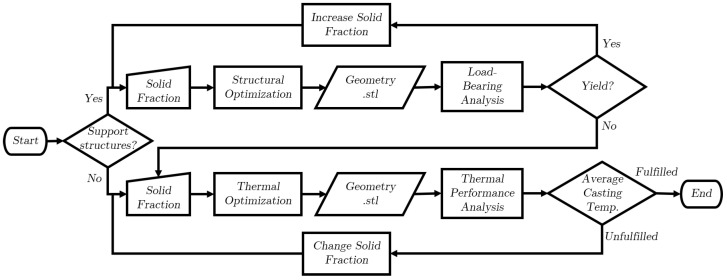
Flowchart of the optimization process.

The optimization process applied within the current investigation is depicted in Figure 2. Initially, a decision is made on whether to generate cooling structures ‘with supports’ or ‘without supports’ depending on the occurring deformations or displacements in the mold. If support structures are needed, the process starts with the input of a solid fraction value. A structural optimization is performed according to the solid fraction on the design domain, generating an ‘.stl’ file of the optimized topology. A load-bearing analysis checks for the von Mises stresses under a defined load acting on the support structures and a decision is made based on yielding whether to increase the solid fraction of the structures and repeat the structural optimization process or jump to the thermal optimization process. The thermal optimization process follows a similar workflow, where the cooling structures are now generated through the thermal Topology Optimization process and the thermal performance of these structures is analyzed by checking the average temperature of the casting and deciding on whether to increase or decrease the solid fraction to further improve the thermal performance or end the process.

### 2.2. Thermal Topology Optimization

Topology Optimization with the density method is a common and straightforward approach to determine the optimal distribution of material in a given design domain for a given objective function and constraints. A design density variable θ is assigned to each finite element, which takes values between 0 (no material) and 1 (solid material) and is associated with physical parameters of the domain such as the thermal conductivity of the material. The problem is solved using the MMA solver with the Topology Optimization Module in COMSOL Multiphysics, which is based on the globally convergent method of moving asymptotes of the form:

(1) $$\begin{array}{c} \mathrm{minimize}\;\;\;f_{0}\left(x\right)\\ \mathrm{subject}\;\mathrm{to}\;\;\;f_{i}\leq 0\;\;\;\;\;\;\;\;\;\;\;\;\;\;\;\;\;\;\;\;\;\;\;\;\;\;\;\;\;i=1,\ldots ,m\\ \;\;\;\;\;\;\;\;\;\;\;\;\;\;\;\;x_{j}^{min}\leq x_{j}\leq x_{j}^{max},\;\;\;\;\;\;\;\;\;\;\;\;\;\;\;j=1,\ldots ,n \end{array}$$

where $f_{0}$ is the objective function, $f_{i}$ are behavior constraints, *m* is the number of constraints, and *x* is a vector of *n* design variables, $x_{j}$. [23]

Steady-state heat transfer is governed by Fourier’s law of heat conduction

(2) −∇·(k∇T)=Q

where *k* is the thermal conductivity and the volumetric heat generation *Q* is proportional to the temperature gradient ∇T.

In the case of pure heat conduction, the objective function $f_{0}$ is minimizing the temperature gradient across the design domain Ω. The Heat Transfer Module was used in COMSOL Multiphysics. Since a material with high thermal conductivity would have a minimal temperature gradient, the problem would be analogical to maximizing the thermal conductivity across the design domain

(3) $$f_{0}=\int_{\Omega }k_{SIMP}{\left(\nabla T\right)}^{2}d\Omega$$

where $k_{SIMP}$ is the thermal conductivity of the material in function of the design density penalized by applying the SIMP rule [10,12]:

(4) $$k_{SIMP}=\left(k_{max}-k_{min}\right)\theta^{n}+k_{min}$$

where $k_{max}$ and $k_{min}$ are the thermal conductivities of the solid material and no material regions, respectively. A ratio of $k_{max}/k_{min}=1000$ is chosen to maximize the heat conduction in regions of solid material and minimize it in regions of no material. The penalization factor is denoted by *n*, where higher values of *n* result in a stricter penalization.

Moreover, a volume constraint is implemented on the solid fraction γ of the domain area to leave space for air to flow around it such as:

(5) $$0\leq \int_{\Omega }\theta d\Omega \leq \gamma$$

In order to obtain mesh-independent results, a Helmholtz filter is used to impose a minimum length scale $R_{min}$ on the domain control variable $\theta_{c}$:

(6) $$\theta_{f}=R_{min}^{2}\nabla^{2}\theta_{f}+\theta_{c}$$

The Helmholtz filter results in large areas with intermediate density values, leading to nonphysical properties of the material. Therefore, a hyperbolic tangent projection is imposed on $\theta_{f}$ to eliminate the intermediate values and obtain sharp boundaries:

(7) $$\theta =\frac{tanh\left(\beta \left(\theta_{f}-\theta_{\beta }\right)\right)+tanh\left(\beta \theta_{\beta }\right)}{tanh\left(\beta \left(1-\theta_{\beta }\right)\right)+tanh\left(\beta \theta_{\beta }\right)}$$

where $\theta_{\beta }$ and β are the projection point and slope, respectively. The values of these parameters are summarized in Table 1.

**Figure 3:**
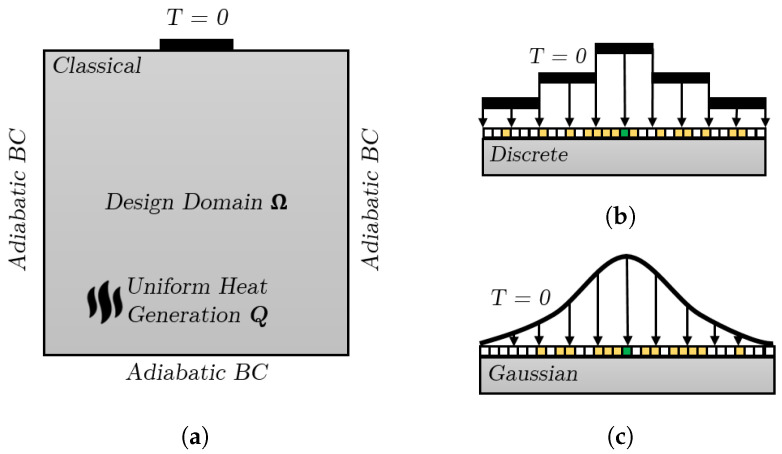
Topology Optimization domains and boundary conditions of three different cases for pure heat conduction. (**a**) Classical. (**b**) Discrete. (**c**) Gaussian.

Figure 3a shows the 2D Topology Optimization domain, which is a standard optimization problem and can be found in the literature [10,11]. All edges of the domain are considered adiabatic, while the temperature of a small portion of one edge is set to T = 0 and a constant uniform heat generation is assumed throughout the domain.

Two new boundary conditions are applied to the edge where the temperature is set to zero in the classical case, as shown in Figure 3b,c. This edge is discretized into several smaller entities (edges), and some of these entities are randomly selected based on a Discrete or a Gaussian probability distribution function, where the temperature is set to T = 0. A MATLAB (version R2023b) script is developed to measure the distance from the center green entity to all other entities and randomly select some of them. This means that entities closer to the center green entity have a higher probability of being selected.

**Figure 4:**
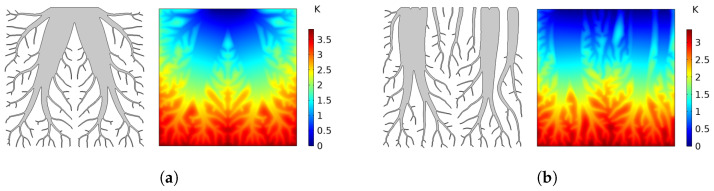
Optimal topology and temperature distributions under different boundary conditions for a solid fraction of SF = 0.3. (**a**) Classical. (**b**) Discrete.

The center green entity is chosen at a location in the casting where larger cooling rates are required, such as castings with thicker cross-sections in some areas. Such a case is already shown in Figure 1, where the middle part of the casting has a thicker cross-section compared to the other parts of the casting. Therefore, the regulation of heat flow in the domain throughout these entities, where the temperature is set to zero, is made possible by this discretization technique as seen in Figure 4. A more gradual and conformal temperature distribution can be seen in the case of a Discrete probability distribution in Figure 4b, compared to a more center-focused temperature distribution in the Classical case in Figure 4a. Cooling structures with a proper temperature distribution regulation can be useful because conformal cooling of the casting requires different cooling rates across different parts of the casting.

**Figure 5:**
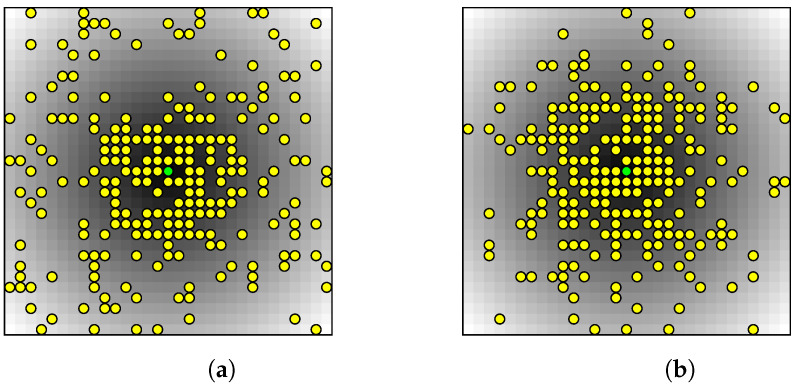
Distance heatmap of randomly selected entities for different probability distribution functions. (**a**) Discrete. (**b**) Gaussian.

The thermal optimization problem is extended to 3D by adjusting the MATLAB script to discretize a face into smaller 2D entities (squares) instead of an edge. Similar to the 2D case, the distance is measured from the center green entity to all other entities by calculating the Euclidean norm and randomly selecting some of these entities (yellow) based on the corresponding probability distribution function, as shown in Figure 5, and set the temperature at these selected entities to zero. All other faces are adiabatic, and a constant uniform heat generation is assumed throughout the 3D domain, which would be a cube in this case. Similarly, entities closer to the green center entity have a higher probability to be selected.

**Figure 6:**
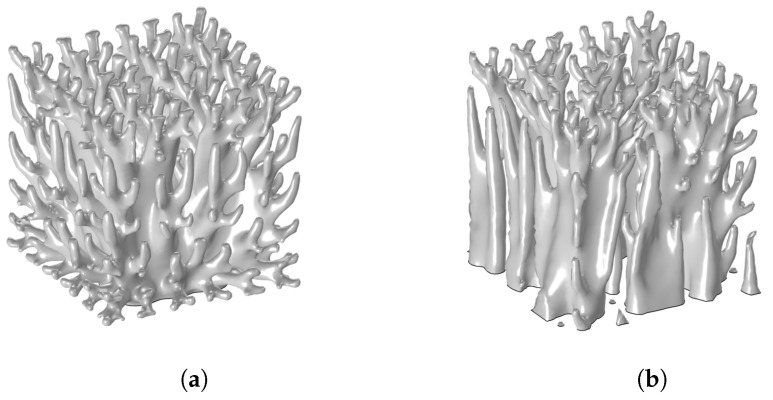
Optimal topology under different boundary conditions for a solid fraction of 30%. (**a**) Classical. (**b**) Discrete.

The generated cooling structures for the Classical and Discrete cases with a solid fraction of SF = 0.3 are shown in Figure 6. It can be noted that the cooling structures are coarser compared to the 2D case since the mesh size and the projection slope were limited.

The 2D Topology Optimization design domain has dimensions of 40 × 40 mm, meshed with a mesh element size of 0.1 mm, leading to 160,000 quadrilateral elements. Table 1 summarizes the parameters of the density model for the 2D and 3D domains. While these values generate decent filtered cooling structures with sharp edges in 2D, they require more computational resources when extended to a 3D domain with dimensions of 40 × 40 × 40 mm. If the same mesh element size of 0.1 mm is chosen, then the domain would be discretized into a total of $64\times 10^{6}$ hexahedral elements, which is a relatively large number of discrete elements to be solved for. Therefore, the total number of mesh elements is restricted to a maximum of 300,000 for the 3D domain. Furthermore, the projection slope is also reduced from 8 to 6, which also decreases the optimization time and facilitates the convergence of the problem. The Topology Optimization problem was solved in parallel on an Intel Xeon W-2295 CPU with 18 cores at 3.00 GHz, where each 2D cooling structure took approximately 100 min to generate, whereas it took each 3D cooling structure approximately 9 h.

### 2.3. Thermomechanical Topology Optimization

The ability of a structure to withstand deformation or deflection in response to an external load is known as structural stiffness. In the case of structural optimization, the optimization problem of choice is the mean compliance, the objective function $f_{0}$ is minimizing the total elastic strain energy, which is equivalent to maximizing the stiffness of a structure [10,24].

**Figure 7:**
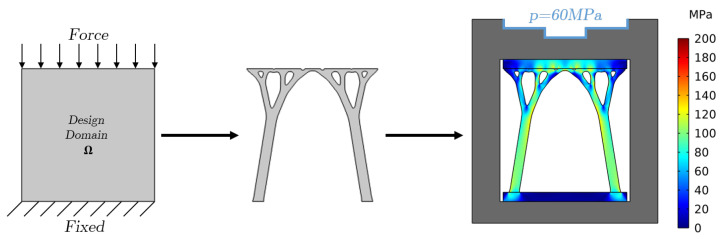
Structural Topology Optimization workflow.

In case supporting structures are required to avoid mechanical failure or deformation, the topology is optimized by fixing one side of the design domain Ω and applying a boundary force on the opposite side, as shown in Figure 7, while also setting a maximum limit constraint for the solid fraction similarly to Section 2.2. The solid fraction of the support structures is fixed to SF = 0.2 in this study. The generated support structures are attached to the die casting mold, and a load-bearing analysis is conducted, where the von Mises stresses are estimated to check for yielding in the support structures by applying a casting pressure on the boundary of the mold (*p* = 60 MPa in this case). It is worth noticing that the von Mises stresses are grayed out (neglected) in the mold since only the stresses in the support structures are of interest.

**Figure 8:**
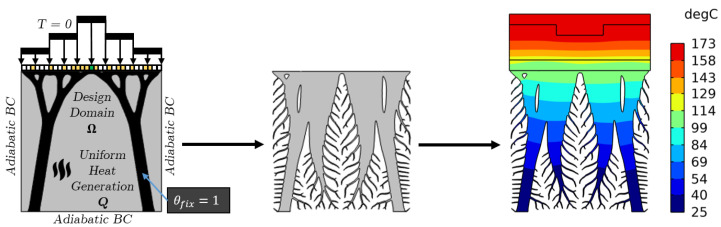
Thermal Topology Optimization workflow.

The support structures are then integrated into the design domain Ω for further thermal optimization. This is performed by fixing the density value $\theta_{fix}=1$ in the design domain, therefore restricting the solver from removing solid material from this region, as shown in Figure 8. Once the thermomechanical cooling structures are generated, they are attached to the casting mold by a base plate to provide a secure connection, and a thermal performance analysis is conducted to assess the performance in terms of lowering the average temperature of the casting and achieving conformal cooling.

### 2.4. Heat Transfer Simulations

**Figure 9:**
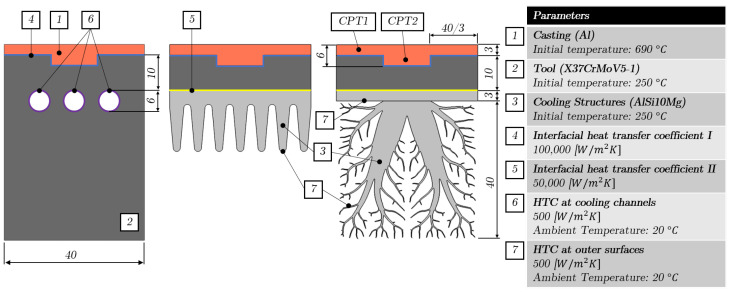
Dimensions, materials, and boundary conditions for all three different setups.

Figure 9 illustrates a simple casting part that utilizes a single cooling structure, while also summarizing the three different setups, domains, materials, and boundary conditions employed in the heat transfer simulations. The first setup consists of three equidistant cooling channels with a diameter of 6 mm that are placed in the casting tool at a distance of 10 mm from the casting. Similarly, fins and cooling structures are added to the casting tool at a distance of 10 mm from the casting in the second and third setups, respectively. Moreover, the fins have a solid fraction of SF = 0.28 compared to the design domain Ω of 40 × 40 mm for the cooling structures. The initial temperature of the casting for pure aluminum is set to T = 690 °C, while the casting tool, fins, and cooling structures are preheated to 250 °C. Cooling is achieved by applying a convective heat transfer coefficient (HTC) to the cooling channels, as well as to both the outer surfaces of the fins and the cooling structures with an ambient temperature of $T_{a}$ = 20 °C. To model the thermal contact resistance between the different domains, interfacial heat transfer coefficients IHTCI and IHTCII are imposed between the corresponding domains, where the surface resistance is reciprocal of the interfacial heat transfer coefficients. Additionally, two casting point temperatures, CPT1 and CPT2, are specified at distinct locations within the casting to evaluate the cooling structures’ effectiveness and their potential impact on shrinkage porosity, as previously outlined in Section 1. Specifically, reducing temperature differentials between CPT1 and CPT2 is critical for minimizing shrinkage porosity formation in the area surrounding CPT2. Therefore, a cooling structure that achieves minimal temperature variations between these two points is considered optimal, aligning with best practices in casting design.

The approximate material properties of the corresponding materials at 250 °C with the relevant units are summarized in Table 2. The material properties of pure aluminum are based on COMSOL’s material database, which are temperature-dependent considering the relatively large variation of temperature in the casting and phase change and do not include Young’s modulus and Poisson’s ratio since they are only used for the thermal simulations. Whereas, the material properties of X37CrMoV5-1 and AlSi10Mg are fixed at specific values, since they do not vary significantly with a relative change in temperature.

The solidification of the casting is modeled by the apparent heat capacity method in COMSOL. The phase change temperature $T_{pc,1\to 2}$, representing the transition from phase 1 (solid) to phase 2 (liquid), is adjusted to accurately reflect the properties of pure aluminum. Accordingly, this temperature is set at the melting point of pure aluminum of 660 °C, with a latent heat $L_{1\to 2}$ of 389 kJ/kg. Although pure aluminum typically exhibits a transition point rather than an interval, a minor transition interval $\Delta T_{1\to 2}$ of 50 °C is introduced for numerical stability. The choice of pure aluminum in this model is deliberate, serving as a representative medium for inserting energy into the system and thereby approximating the behavior of various casting alloys. The Heaviside phase transition function, in conjunction with the transition interval, facilitates a smooth material transition between phases in the simulation.

## 3. Results and Discussion

### 3.1. Load-Bearing Analysis

**Figure 10:**
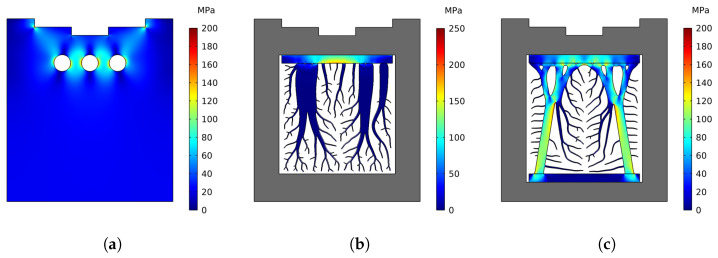
Von Mises stresses for a casting pressure of 60 MPa and with a solid fraction of SF = 0.3 for the cooling structures. (**a**) Cooling channels. (**b**) Discrete. (**c**) Discrete with support structures.

A load-bearing analysis was conducted on a die casting mold with cooling channels, where the Von Mises stresses were compared to a mold with different types of cooling structures. The numerical results for the load-bearing analysis of the cooling channels and cooling structures with and without supports with a solid fraction of SF = 0.3 are shown in Figure 10. The results are interpreted for a casting pressure of 60 MPa, applied at the boundaries of the casting, and the von Mises stresses are illustrated. It is observable that the stress concentrations are located around the cooling channels, as seen in Figure 10a, where the maximum von Mises stress was found to be 200 MPa. Hence, special care should be taken when placing the cooling channels in the mold in order to avoid any deformations around the channels. The stress concentrations are focused locally within a specific region just below the castings part in case of cooling structures without supports, as illustrated in Figure 10b, where the von Mises stresses reached a maximum of 250 MPa. On the other hand, when supports are included in the cooling structures, the stress concentrations are well distributed along the support structures, as observed in Figure 10c, while also reducing the maximum von Mises stresses to a maximum of 200 MPa, similar to that of the cooling channels, therefore reducing local deformations within the mold and the cooling structures.

**Figure 11:**
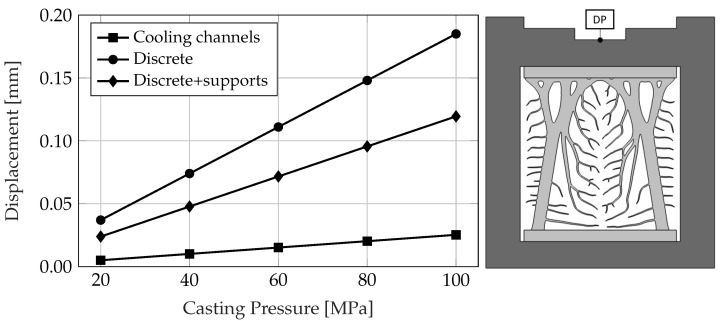
Displacement magnitude of the Displacement Point (DP) under different casting pressures for different cases.

Additional load-bearing simulations, utilizing varying casting pressures, were carried out to assess the local displacement at a specific Displacement Point (DP) within the die casting mold. This point was identified as the location most susceptible to deformation, as indicated in Figure 11. As anticipated, the simulations indicate that displacement is minimized with the use of cooling channels and maximized in cooling structures lacking supports (referred to as “Discrete”). However, the introduction of support structures into the cooling structures’ design significantly reduces displacement. Thus, cooling structures equipped with supports are deemed more beneficial compared to those without, taking into account the constraints associated with the Displacement Point (DP) or deformations within the casting. Yet, it is still necessary to define an acceptable threshold for displacement and stress concentration applicable to the geometry of an actual die casting part.

### 3.2. Thermal Performance Analysis

**Figure 12:**
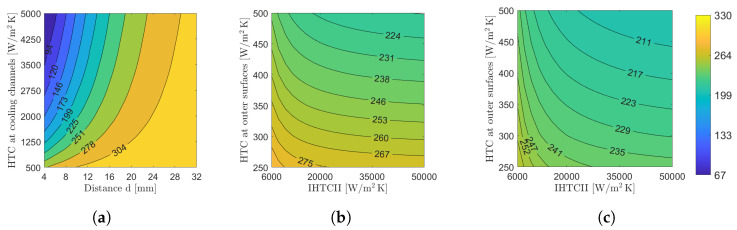
Parametric study of the average temperature of casting $T_{avg}$ for the three different setups under different thermal boundary conditions (see Figure 9 for the description of the parameters). (**a**) Cooling channels. (**b**) Fins. (**c**) Classical cooling structure with a solid fraction of SF = 0.3.

A parametric study was carried out on three distinct setups to offer a preliminary insight into their expected thermal behavior. These setups are depicted in Figure 9, while the outcomes, including the average temperature of casting $T_{avg}$, are showcased in Figure 12. Specifically, in Figure 12a, the heat transfer coefficient HTC at the cooling channels and the distance of these channels from the casting were varied with increments of 125 W/m^2^K and 2 mm, respectively. It can be seen that the average temperature of casting in the parametric study of the cooling channels experiences notable variations, especially with respect to the distance from the casting, therefore correct positioning of the cooling channels without being subject to severe deformations is crucial to the integrity and thermal performance of the die casting mold. Moreover, the impact of different heat transfer coefficients on the average temperature of casting is noteworthy, especially for lesser values, hence a reduction in the heat transfer coefficient due to gradual development of limescale, dirt, and corrosion, as mentioned in Section 1, also remains crucial. Similarly, the heat transfer coefficient at the outer surfaces and the interfacial heat transfer coefficient II of the fins and the classical cooling structure were varied with increments of 50 and 2000 W/m^2^K, respectively, in Figure 12b,c. Likewise, significant variations in the average temperature of casting can be seen in the parametric study of the fins and the classical cooling structure, primarily with respect to the heat transfer coefficient at the outer surfaces. Consequently, this highlights the estimation difficulty of an adequate heat transfer coefficient, since regions with flow recirculation will diminish the local heat transfer rate as previously mentioned in Section 1. Despite the initial lack of precise knowledge regarding the exact values for heat transfer coefficients and distances, the contour plots reveal that setups in Figure 12b,c exhibit beneficial properties across a broad spectrum of parameters. The outcomes associated with the setup in Figure 12a are attained only through meticulous temperature control via contours, necessitating high heat transfer coefficients between the mold wall and the fluid.

**Figure 13:**
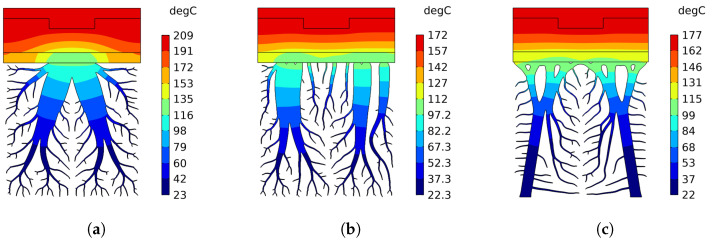
Temperature contours for a solid fraction of SF = 0.3 after a cooling of 30 s. (**a**) Classical. (**b**) Discrete. (**c**) Discrete with support structures.

The topology-optimized cooling structure depicted in Figure 9 illustrates an initial design not yet specifically tailored to the requirements of the intended casting component. To address this, heat transfer simulations of the generated topologies were performed under various boundary conditions. This process aimed to explore and assess the thermal performance limits, ensuring the design’s effectiveness for its intended application. The temperature contours of three different cooling structures in 2D for a solid fraction of SF = 0.3 after a total cooling time of 30 s are shown in Figure 13. It can be seen that the heat transfer occurs via the main branch of the cooling structure for the Classical case, causing a congestion of the heat flow. On the other hand, the Discrete case with and without supports exhibits a more proportional temperature gradient across all domains in the vertical direction, as already discussed in Section 2.2. Moreover, the highest temperature value is lower for the Discrete case, suggesting a better cooling performance compared to the classical case.

**Figure 14:**
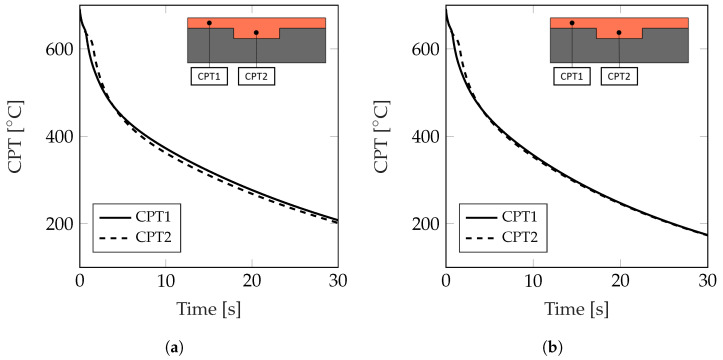
Variation in the casting point temperatures CPT1 and CPT2 for a solid fraction of SF = 0.3. (**a**) Classical. (**b**) Gaussian.

Figure 14 shows the variation in the casting point temperature CPT1 and CPT2 for the Classical and Gaussian cases without support structures for a solid fraction of SF = 0.3 and a total cooling time of t = 30 s. For the Classical case, CPT1 experiences initially a higher cooling rate since its temperature decreases faster than that of CPT2, but the opposite is observed for the remaining of the cooling process, where CPT2 has a lower temperature than CPT1, which leads to non-conformal cooling. A conformal cooling profile is observed in the Gaussian case, where the temperature profiles of CPT1 and CPT2 are aligned to each other, after an initial cooling of approximately t = 4 s, for the rest of the cooling process.

**Figure 15:**
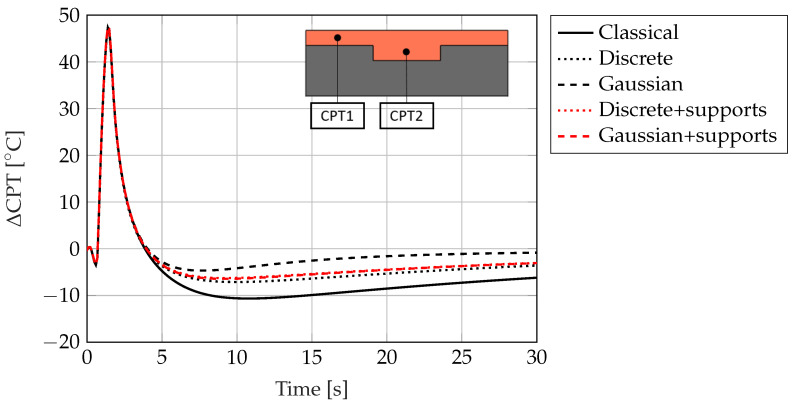
Temperature difference ΔCPT=CPT2−CPT1 for different boundary conditions and for a solid fraction of SF = 0.3.

Additional studies are performed to further assess the capabilities of the different Topology Optimization cases under different boundary conditions in Figure 15. For a solid fraction of SF = 0.3, the difference in casting point temperatures ΔCPT is calculated for multiple cases during a total cooling time of t = 30 s. It can be seen that different boundary conditions exhibit different ΔCPT profiles, which reaches its maximum during the initial stage of the cooling process. Notably, the cooling structures with Discrete and Gaussian boundary conditions show better uniform cooling than the Classical case. Significant improvement can be seen in the Gaussian case without supports, where these two casting points are cooled down to almost the same temperature after a total cooling time of t = 30 s, while keeping a conformal cooling profile for the time interval 20 < t < 30 s. All other cases experience a similar cooling profile between the time interval 5 < t < 30 s, where they reach a ΔCPT of 5 °C at t = 30 s.

**Figure 16:**
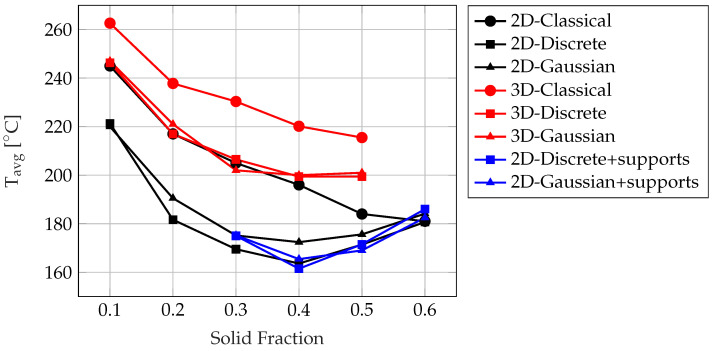
Average temperature of casting $T_{avg}$ under different boundary conditions and solid fractions for 2D and 3D cases.

The average temperature of casting $T_{avg}$ after 30 s of cooling of the cooling structures under different boundary conditions and solid fractions for 2D and 3D cases are summarized in Figure 16. In the cases where cooling channels and fins were used for the cooling of the die casting mold, the average temperatures of casting $T_{avg}$ were 304 °C and 216 °C, respectively. When compared to the cooling channels, practically all examples showed a notable improvement in the casting’s cooling, where the highest $T_{avg}$ was found to be 262 °C for the 3D-Classical case with the lowest solid fraction of SF = 0.1, and the lowest $T_{avg}$ was found to be 199 °C for the 3D-Discrete case with a solid fraction of SF = 0.4. Notably, the 3D cooling structures were all outperformed by the 2D cooling structures in all different cases and solid fractions and the fins. This can be due to the fact that the mesh size and the projection slope used in the 3D Topology Optimization were limited to avoid relatively long Topology Optimization times, as already discussed in Section 2.2, thus resulting in coarser cooling structures where its total surface area is much lower compared to the 2D cases. Concerning the 2D cooling structures, although the Classical case performed poorly when evaluated against the fins for solid fractions of 0.1 and 0.2, a superior cooling was achieved when using the Discrete and Gaussian boundary conditions for solid fractions ranging from 0.2 to 0.6 both with and without supports, where the lowest $T_{avg}$ was found to be 161.5 °C for the Discrete boundary conditions with supports with a solid fraction of SF = 0.4. Noteworthy, better cooling was achieved with a lower solid fraction with the Discrete and Gaussian boundary conditions compared to SF = 0.28 of the cooling fins, as already mentioned in Section 2.4. Hence, there are benefits to appropriate material distribution in the optimization domain under effective boundary conditions. Additionally, there is an optimal solid fraction for each case around a solid fraction of SF = 0.4; a greater solid fraction does not necessarily result in the minimization of the average temperature of the casting. It is worth noting that the addition of supports to the 2D cooling structures did not introduce any drawbacks in the thermal optimization process. Instead, the thermal performance remained almost identical when compared to cases without supports, particularly for the Discrete and Gaussian boundary conditions with solid fractions of 0.4 and 0.5. This outcome was achieved while retaining all the benefits derived from maintaining the structural integrity of the die casting mold.

**Figure A1:**
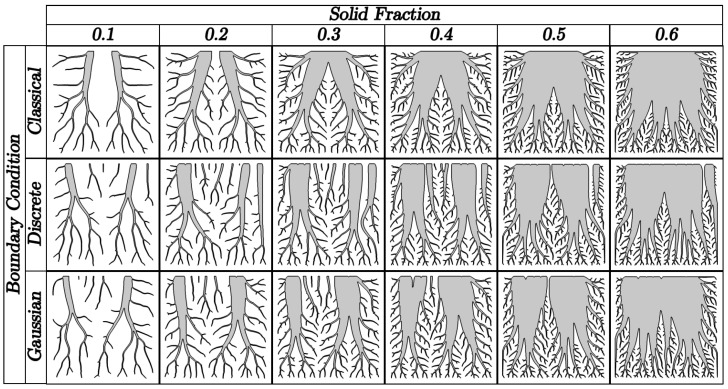
Generated 2D cooling structures without supports.

Appendix A contains the figures of all the generated cooling structures for all different cases. Figure A1 shows the geometries generated in 2D under Classical, Discrete, and Gaussian boundary conditions without supports for solid fractions of 0.1 < SF < 0.6, whereas the solid fraction for the cases with supports was restricted to 0.3 < SF < 0.6 in order to accommodate for the mechanical supports. Moreover, a solid fraction range of 0.1 < SF < 0.5 was used for the 3D case.

## 4. Conclusions

In this paper, an enhanced replacement was sought for the conventional cooling channels used in die casting molds, which were demonstrated to perform poorly, especially with the formation of dirt, limescale, and corrosion. Topology Optimization was introduced as a method of augmenting the thermal performance in terms of cooling the die casting mold. Enhanced cooling also enables replacement of the coolant with air, which solves problems such as limescale and dirt formation. Different cooling structures under different boundary conditions and with different solid fractions were generated and simulated for their thermal and structural performance. The Topology Optimization Module with the MMA solver and the Heat Transfer Module were used in COMSOL Multiphysics. A parametric study was conducted where the influence of different parameters on the average temperature of casting $T_{avg}$ such as the distance of the cooling channels to the casting and the heat transfer coefficient at the outer surfaces of the fins and the cooling structures was highlighted. Discrete and Gaussian probability distribution functions were proposed as two new boundary conditions for the Topology Optimization problem, where superior and conformal cooling was achieved compared to the conventional cooling channels, the fins, and the Classical Topology Optimization case in 2D by evaluating the average casting temperature $T_{avg}$ and the casting point temperatures CPT1 and CPT2. Another case was simulated where the cooling structures were optimized both thermally and mechanically by including support structures within the design domain. It was demonstrated that the addition of support structures into the optimization domain resulted in reduction of the displacement at a certain Displacement Point (DP) located at a position in the mold where high deformation is expected due to the casting pressure. Moreover, the cooling structures with supports demonstrated a similar thermal performance compared to the structures without supports in the case of Discrete and Gaussian probability density functions while keeping the benefits a better structural integrity of the die casting mold. Concerning the 3D thermal optimization case, the cooling structures revealed enhanced cooling when compared to the conventional cooling channels but failed to demonstrate any additional performance in contrast to the 2D cooling structures and fins. The main limitation of the 3D cooling structures was the relatively long computational time required to generate the geometries with a fine mesh; therefore, the mesh size was limited to a maximum number of mesh elements, resulting in coarser structures when compared to the 2D case. The impact of different local heat transfer coefficients must be comprehended in depth; this can be achieved by the implementation of computational fluid dynamics (CFD) and conjugate heat transfer (CHT) simulations in order to analyze the recirculation zones and the local heat transfer rate across different parts of the cooling structures. More complex casting geometries (especially in 3D) should also be further investigated to determine the true performance of these cooling structures, since the casting’s geometry was kept fairly simple and symmetrical in this study. Furthermore, the permissible dimensions of the cooling structures within the die casting tool (see Figure 1b) introduce an additional parameter to the optimization process. This parameter is worth thorough investigation due to its substantial impact on the cooling performance of the structures. The objective should be to identify an optimal balance between the allowable space for these structures and the resultant cooling efficiency. Accurate and reliable manufacturability of the cooling structures should be further investigated either by traditional manufacturing methods such as investment casting or modern methods such as additive manufacturing techniques. A holistic approach, potentially integrating both traditional and modern methods, could offer comprehensive insights into optimal fabrication strategies of these structures.

## Figures and Tables

**Table 1:** Topology Optimization parameters for 2D and 3D domains.

Domain	Mesh Size (mm)	$R_{\min}$	β	$\theta_{\beta }$	$P_{\mathrm{SIMP}}$	$\theta_{\min}$	$k_{\max}$	$k_{\min}$
2D	0.1	2 × meshsize	8	0.5	3	0.001	1	0.001
3D	0.6	2 × meshsize	6	0.5	3	0.001	1000	1

**Table 2:** Material properties at 250 °C of pure aluminum, X37CrMoV5-1, and AlSi10Mg used in the numerical simulations. Density ρ, thermal conductivity *k*, specific heat capacity $C_{p}$, Young’s modulus *E*, and Poisson’s ratio ν.

Material	ρ [kg/m^3^]	*k* [W/(m · K)]	$C_{p}$ [J/(kg· K)]	*E* [GPa]	ν
Al	COMSOL	COMSOL	COMSOL	N/A	N/A
X37CrMoV5-1	7716	28.7	511.5	171.8	0.3
AlSi10Mg	2640	115	968	65	0.33

**Figure A2:**
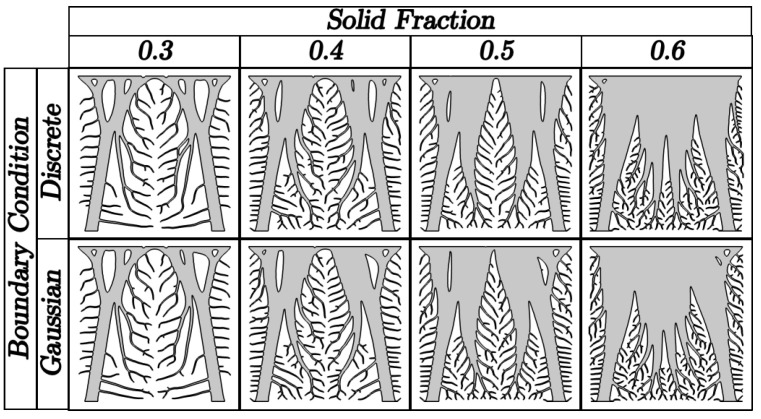
Generated 2D cooling structures with supports.

**Figure A3:**
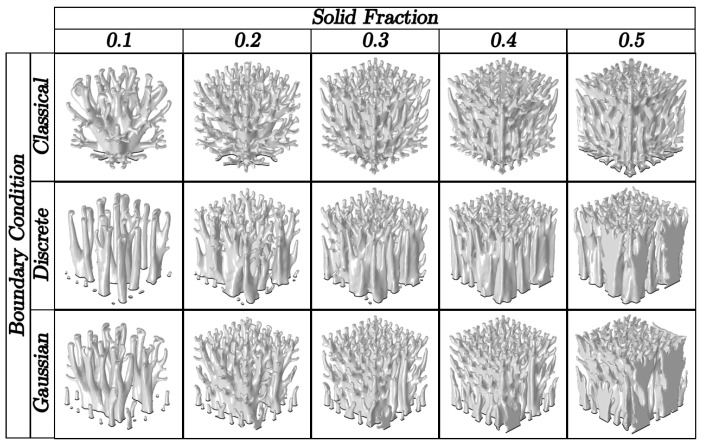
Generated 3D cooling structures.

## Data Availability

The data presented in this study are available on request from the corresponding author (the data are not publicly available due to privacy).
